# Unraveling Shallot Viral Diversity: PCR Detection and In Vitro Culture

**DOI:** 10.3390/pathogens15080799

**Published:** 2026-07-28

**Authors:** Kelly Zúñiga-Vera, Martina Albuja-Quintana, Diana Calderón, Miguel Orellana, Carlos Ruales, Maria de Lourdes Torres

**Affiliations:** 1 Laboratorio de Biotecnología Vegetal, Colegio de Ciencias Biológicas y Ambientales, Universidad San Francisco de Quito, Quito 170901, Ecuador; 2 Instituto de Microbiología, Colegio de Ciencias Biológicas y Ambientales, Universidad San Francisco de Quito, Quito 170901, Ecuador; 3 Laboratorio de Biotecnología Agrícola y de Alimentos, Colegio de Ciencias E Ingenierías—Ing. Agronomía, Universidad San Francisco de Quito, Quito 170901, Ecuador

**Keywords:** *Allium cepa* var. *aggregatum*, shallot latent virus, Iris yellow spot virus, molecular detection, shoot tips, in vitro culture

## Abstract

Shallot (*Allium cepa* var. *aggregatum*) is an annual herbaceous plant of the Amaryllidaceae family cultivated worldwide for its gastronomic importance, unique flavour, and nutritional properties. In Ecuador, shallot is considered an emerging crop; however, the lack of effective detection and treatment options for phytoviruses remains a challenge for farmers. These viruses negatively impact crop yield, leading to substantial economic losses and declining crop quality. Simultaneous infections can synergistically exacerbate crop damage. In this study, the molecular detection of the five most common shallot viruses—Shallot latent virus (SLV), Onion yellow dwarf virus (OYDV), Shallot virus X (ShVX), Leek yellow stripe virus (LYSV), and Iris yellow spot virus (IYSV)—were investigated in shallots obtained from different market suppliers in Quito, Ecuador. We identified two viruses using RT-PCR: SLV (latent) and IYSV (responsible for severe disease symptoms). Shoot tip culture produced shallot plants completely free of IYSV, while SLV persisted in the regenerated plants. This study reports IYSV for the first time in shallots in Ecuador and shows that shoot tip culture can serve as a partial cleaning method to improve crop quality and support more effective pathogen management and control strategies.

## 1. Introduction

Shallot (*Allium cepa* var. *aggregatum*), a distinct variety within the *Allium cepa* species commonly known as onions, is an annual herbaceous plant of the Amaryllidaceae family [1]. It is widely cultivated across the Americas, Europe, and Asia [2]. This variety, known for its vegetative reproduction via bulbs, is distinguished by its extended storage life, colour, and bioactive compounds [3,4]. Shallots are well known in the culinary world as an essential ingredient in various dishes due to their unique flavour and nutritional properties [5]. They are a rich source of valuable phytochemicals, including organosulfur compounds, fructans, and flavonoids [1,6]. However, they are highly susceptible to pests and diseases, with viral infections among the most severe threats to yield and quality [7,8]. Viruses account for nearly half of emerging infectious diseases in various crops and are responsible for approximately 40% of total crop losses [9]. Several economically important viral pathogens from the *Allexivirus*, *Carlavirus*, *Potyvirus,* and *Tospovirus* genera frequently occur in infections that can further exacerbate yield reduction and crop losses [7,10]. The development and severity of these viral diseases depend on several factors, including the plant’s age, environmental conditions, and the vector dynamics [11].

Viral pathogens can usually be detected using serological tests, bioassays, or DNA- and RNA-based molecular techniques [12]. Bioassays involve grafting plant material or mechanical inoculation onto a virus indicator host, with symptoms typically developing within days to a few weeks in herbaceous plants and over a longer period in woody plants [13,14,15]. Serological methods are based on the specific interaction between viral antigens and antibodies. However, producing highly specific antisera against *Allium* viruses is challenging due to coinfections [16,17], and the presence of phenolic compounds in some plants [18,19] interfere with the isolation of active viruses required for antiserum preparation. Monoclonal antibodies have been utilized to overcome this problem and differentiate between strains of potyviruses and carlaviruses by using the variability of the N-terminal viral coat protein (CP) reaction [18,20]. Molecular methods for viral nucleic acid detection have been optimized and developed for large-scale virus testing of shallot leaves and bulbs. Among these, Polymerase Chain Reaction (PCR) and Reverse Transcription PCR (RT-PCR) provide specific, versatile, and sensitive tools for virus diagnosis [21,22]. These techniques involve primer design based on specific conserved sequences of viral genetic material, resulting in varying levels of sensitivity and specificity [7,23].

While such diagnostic tools are essential for early detection, controlling viral infection in plants requires a comprehensive approach. Several virus management strategies have been reported, including the use of certified virus-free seeds, control of arthropod vectors, the use of insecticides, proper field management, the removal of infected plants, the use of resistant varieties, virus management during post-harvest, crop rotation, surveillance, and monitoring [7,24,25,26,27]. Current plant protection strategies include genome editing, marker-assisted selection, and RNA silencing technologies, which provide effective genetic protection [28,29,30]. However, these approaches are often virus-specific, short-lived, and may promote gene silencing and shifts in virus populations [31]. Virus inhibitors can suppress viral infections, but they may also cause phytotoxicity and teratogenicity [32]. The use of elicitors to induce systemic resistance is environmentally friendly and biosafe, but its efficiency is low [32,33]. Chemical control is ineffective against intracellular pathogens such as viruses [34]. Vector control is widely used as a preventive measure, but its high cost, limited effectiveness, and negative environmental impacts constrain its application [25]. More recently, beneficial microorganisms and microbial enzymes have shown promise for controlling vector-borne viruses and reducing the use of chemical pesticides; however, their efficacy under field conditions remains limited [25,32]. Advances in nanotechnology, particularly nanoliposomal formulations of plant-derived biostimulants and biopesticides, have improved plant growth and tolerance to abiotic and biotic stresses, including antibacterial, antifungal, antiviral, and insecticidal effects. At the same time, these lipid-based systems enhance bioactivity, stability, and target specificity. However, challenges related to scalability, regulatory compliance, and cost-effectiveness continue to limit their widespread adoption [35].

One widely used strategy for propagating virus-free plants in *Allium* crops is through meristem or shoot tip culture [36,37,38]. This method is particularly effective due to its high genetic stability and regeneration potential [7,12,36,39,40,41,42]. Shoot tip culture is a type of meristem culture in which the explant consists of the shoot tip, including the apical meristem and a small number of surrounding leaf primordia [43]. Notably, this method has been reported to be an efficient tool for obtaining virus-free plants while maintaining a high regeneration rate [43,44]. In *Allium* species, virus elimination efficiencies of approximately 50–70% and even higher have been reported depending on the virus [36,45,46].

In Ecuador, shallots are considered an emerging crop [47]. However, the lack of effective detection and treatment options for viral infections remains a significant challenge for farmers [9]. Virological research in Ecuador focuses on export crops (banana, cocoa, and coffee), staple foods in the national diet (potato, maize, and rice), and high-value fruit crops (tree tomato, cucurbits, and papaya), because of their relevance to the economy and food security [48,49,50]. Diagnostic efforts have relied on immunological and molecular techniques, such as DAS-ELISA and PCR [51]. Although shallots are infected by various viruses, the five most common viruses reported worldwide when this study began in 2022 were Shallot latent virus (SLV), Onion yellow dwarf virus (OYDV), Shallot virus X (ShVX), Iris yellow spot virus (IYSV), and Leek yellow stripe virus (LYSV) [52,53,54].

Viral detection and its elimination are essential for sustainable crop production through vegetative propagation strategies [9,12]. Based on previous reports of viral infections in *Allium* crops in Ecuador and the successful use of meristem and shoot tip for virus sanitation, we hypothesized that molecular methods would enable the detection of major viruses infecting shallots and that implementing shoot tip culture could effectively eliminate these viruses, enabling the successful regeneration of virus-free plantlets. The objectives of this study were (1) to detect the five most common viruses infecting shallots using molecular methods—Shallot latent virus (SLV), Onion yellow dwarf virus (OYDV), Shallot virus X (ShVX), Leek yellow streak virus (LYSV), and Iris yellow spot virus (IYSV) [7,55]—and (2) to evaluate the effectiveness of shoot tip culture in eliminating viruses present in shallots (*Allium cepa* var. *aggregatum*) and producing virus-free plants [56].

## 2. Materials and Methods

This study is based on the master’s thesis of Zúñiga-Vera [56].

### 2.1. Design and Selection of Primers

Five of the most common viruses affecting shallots were selected for molecular detection by RT-PCR: OYDV, SLV, ShVX, IYSV, and LYSV. Nucleotide sequences of the target genes of the viruses were obtained from the NCBI: https://www.ncbi.nlm.nih.gov/ (accessed on 26 July 2024). The target sites selected were the coat protein for SLV, OYDV, and LYSV, and the replicase and nucleocapsid genes for ShVX and IYSV, respectively. Sequences were downloaded in FASTA format and aligned in UGENE (v45.0). Primers were designed using Primer3web (v4.1.0) [57] and validated with PrimerBLAST: https://www.ncbi.nlm.nih.gov/tools/primer-blast/ (accessed on 26 July 2024) [58] and MFEprimer (v3.1) [59]. Because sequence variations existed, degenerate primers were designed. Two sets of primers were tested: primers designed in this study and those previously reported in the literature (Table S1) [7,60,61,62]. Based on the analysis of the PCR results from pilot shallot samples, literature-based primers were selected for SLV and OYDV. In contrast, designed primers were chosen for ShVX, IYSV, and LYSV for molecular analysis (Table S2 [60,61] and Figures S1 and S2).

### 2.2. Synthetic DNA-Based Positive Controls for RT-PCR Detection of Plant Viruses

The target sequences for each virus (nucleocapsid for IYSV, replicase for ShVX, and coat protein for SLV, OYDV, and LYSV) were obtained from NCBI. Small fragments between 269 and 625 bp of the target sequences were selected for cloning into an ampicillin plasmid vector (pMG-Amp) and evaluated through an in silico analysis. The expected sizes of PCR products were SLV (308 bp), OYDV (625 bp), ShVX (160 bp), IYSV (189 bp), and LYSV (191 bp) (Figure S1).

### 2.3. Plant Material for Viral Detection and In Vitro Culture

**Figure 1:**
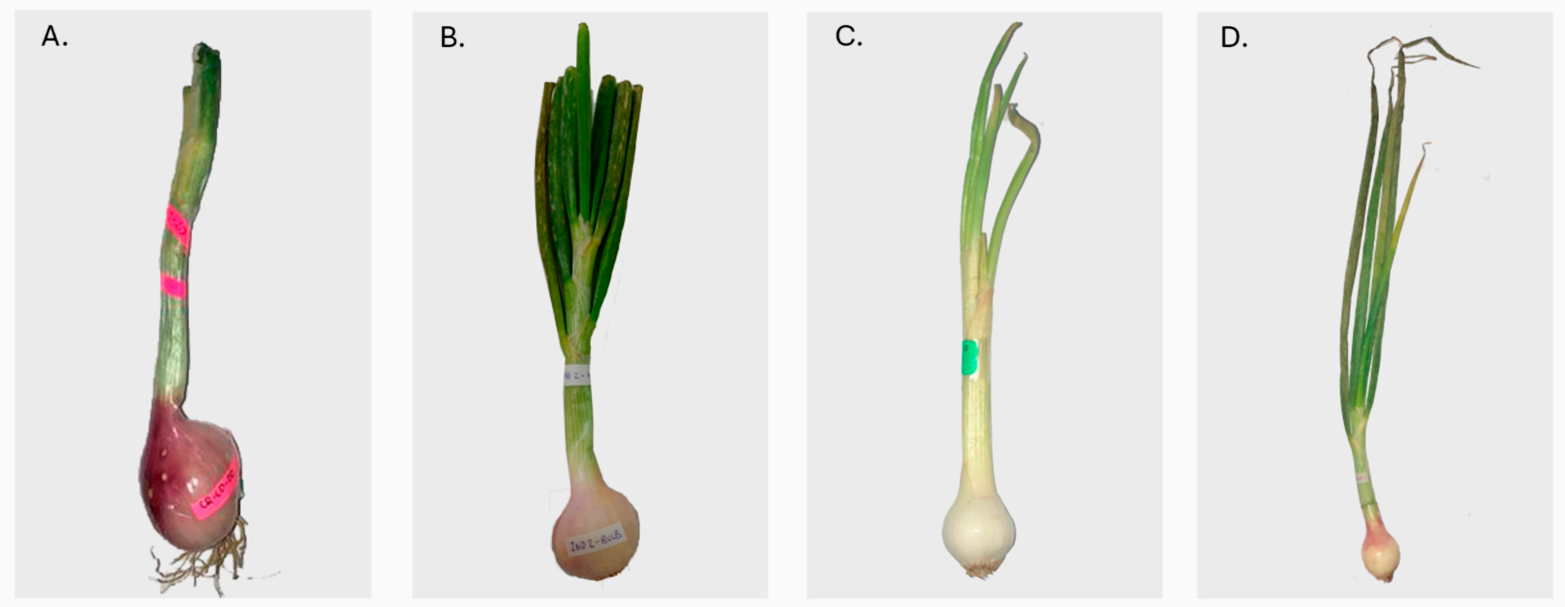
Plant material. (**A**) Experimental farm. (**B**) Organic market. (**C**) Iñaquito market 1. (**D**) Iñaquito market 2. Adapted from [56].

Between 2022 and 2023, a total of 99 shallot plants (*Allium cepa* var. *aggregatum*) were obtained from three different suppliers based on availability (Figure 1): 23 samples originated from the Experimental Farm of Universidad San Francisco de Quito (EF), located in Tumbaco, Pichincha (2400 m a.s.l.; 78°24′ W, 00°23′ S); 26 samples obtained from the organic market (OM) corresponded to products sold by an agroecological company located in Amaguaña, Pichincha, at elevations ranging from 2400 to 2600 m a.s.l. The crops are managed under certified organic production systems, without synthetic pesticides or fertilizers. This region is characterized by volcanic soils and a temperate climate, conditions that are favourable for agricultural production [63,64]. The remaining 50 samples were obtained from two suppliers at the Iñaquito market in Quito (Iq1 and Iq2), who sell shallots produced in the provinces of Chimborazo (2300–2600 m a.s.l) and Tungurahua (1815–3203 m a.s.l), both located in the Ecuadorian Andes. These regions are characterized by inter-Andean valleys and highlands, a temperate to cold climate, predominantly volcanic soils with high fertility and organic matter content, and diversified agricultural systems [65,66,67]. Production is carried out mainly under intensive and semi-intensive farming systems, with irrigation used in some areas and management practices adapted to the local climatic conditions [65,68]. The plants were transported to the Plant Biotechnology Laboratory at USFQ. Bulbs were separated from the leaves and stored at room temperature (18 °C) for in vitro culture. Meanwhile, the leaves were stored at –20 °C for 24 h and then at –80 °C for viral detection.

### 2.4. RNA Extraction from Shallot Leaves Pre and Post Culture

Virus detection was carried out both before and after in vitro culture. Pre-culture detection aimed to identify viruses present in the original material, while detection after in vitro culture assessed the effectiveness of shoot tip culture in eliminating them. Before in vitro culture, RNA was extracted from all 99 shallot leaf samples. After the shoot tip culture, only 31 plantlets were available for molecular analysis: 11 from EF (Assay 1), 8 from Iq1 (Assay 2), and 12 from Iq2 (Assay 3). Total RNA was extracted from leaves using the TRIzol-chloroform protocol as described by Rio et al. [69] with approximately 200–300 mg of shallot leaf powder per sample. RNA quantity and purity, as well as integrity, were checked by Nanodrop (Thermo Fisher Scientific, Waltham, MA, USA) and Qubit (Invitrogen, Carlsbad, CA, USA). After extraction, the RNA was left at 4 °C for 24 h and then stored at −20 °C.

### 2.5. RT-PCR and Sanger Sequencing

Complementary DNA (cDNA) was synthesized using the SuperScript III Reverse Transcriptase (Invitrogen, Carlsbad, CA, USA) and random hexamer primers (Thermo Fisher Scientific, Waltham, MA, USA), following the manufacturer’s instructions. PCR was then performed using virus-specific primers, along with synthetic positive and negative controls. The PCR cycling conditions were 94 °C for 2 min, followed by 30 cycles of 94 °C for 30 s, 58 °C (OYDV, LYSV), 47 °C (SLV), 61.5 °C (ShVX), and 62 °C (IYSV) for 30 s, 72 °C for 1 min, and finally an extension of 72 °C for 3 min. RT-PCR products were visualized in a 1.5% agarose gel, stained with SYBR Safe (Invitrogen, Carlsbad, CA, USA), and examined under UV light using a photo-documenter (Bio-Rad Laboratories, Hercules, CA, USA). Three PCR amplicons for each virus were selected and submitted for Sanger sequencing. The resulting sequences were processed in SnapGene (v8.2.2) and MEGA (v10.2.4), and the consensus sequences obtained in Geneious (v11.0.9) were compared with those available in the NCBI GenBank database using the online BLASTn: https://blast.ncbi.nlm.nih.gov/Blast.cgi (accessed on 10 July 2026) to confirm the identity of each virus isolate. These sequences were uploaded to the GenBank (NCBI). Analytical validation parameters, such as the limit of detection (LOD) and assay sensitivity, were not experimentally determined.

### 2.6. Bulb Sterilization

The in vitro culture protocol used in this study was standardized and adapted from previously developed protocols [47,70], with some modifications detailed in Table S3 and Figure S3. These modifications included using fresh bulbs without a drying or cooling period to improve shoot initiation and regeneration, replacing Benomyl (Agripac, Guayaquil, Ecuador) with Mertect (Syngenta, Basel, Switzerland) due to regulatory restrictions on Benomyl in Ecuador, incorporating a fungicide-based bulb disinfection step, and excluding fungicides from all culture media to prevent adverse effects on shoot tip growth. A total of 73 fresh shallot bulb samples were sterilized: 23 from EF (Assay 1), 25 from Eq1 (Assay 2), and 25 from Eq2 (Assay 3). The OM samples were excluded from the in vitro assays due to fungal contamination. Assay 1, Assay 2, and Assay 3 represent three independent experimental runs established on different dates under identical culture conditions. The disinfection protocol began with a 10 min pre-wash of the bulbs in tap water to remove soil residues and outer dry layers. The bulbs were then immersed in Mertect fungicide (3.5 g/L) and agitated at 420 rpm for 15 min.

Afterward, in a laminar flow chamber (Labconco Purifier Clean Bench; Labconco Corporation, Kansas City, MO, USA), bulbs were immersed in ethanol (70%) for 10 min. Then, the bulbs were submerged in sodium hypochlorite (4.5%) with three drops of Tween 20 (Sigma-Aldrich, St. Louis, MO, USA) and agitated for 35 min. The bulbs were then rinsed with sterile distilled water. One-third of the length of the bulb was excised with a sterile scalpel, retaining the basal portion and removing external cataphylls to obtain a basal disc explant of about 1.5 cm in diameter and 1 cm in height. Each explant was cultured on M1 initial medium in a glass bottle (Table S4) [47].

### 2.7. Shoot Tip Culture

**Figure 2:**
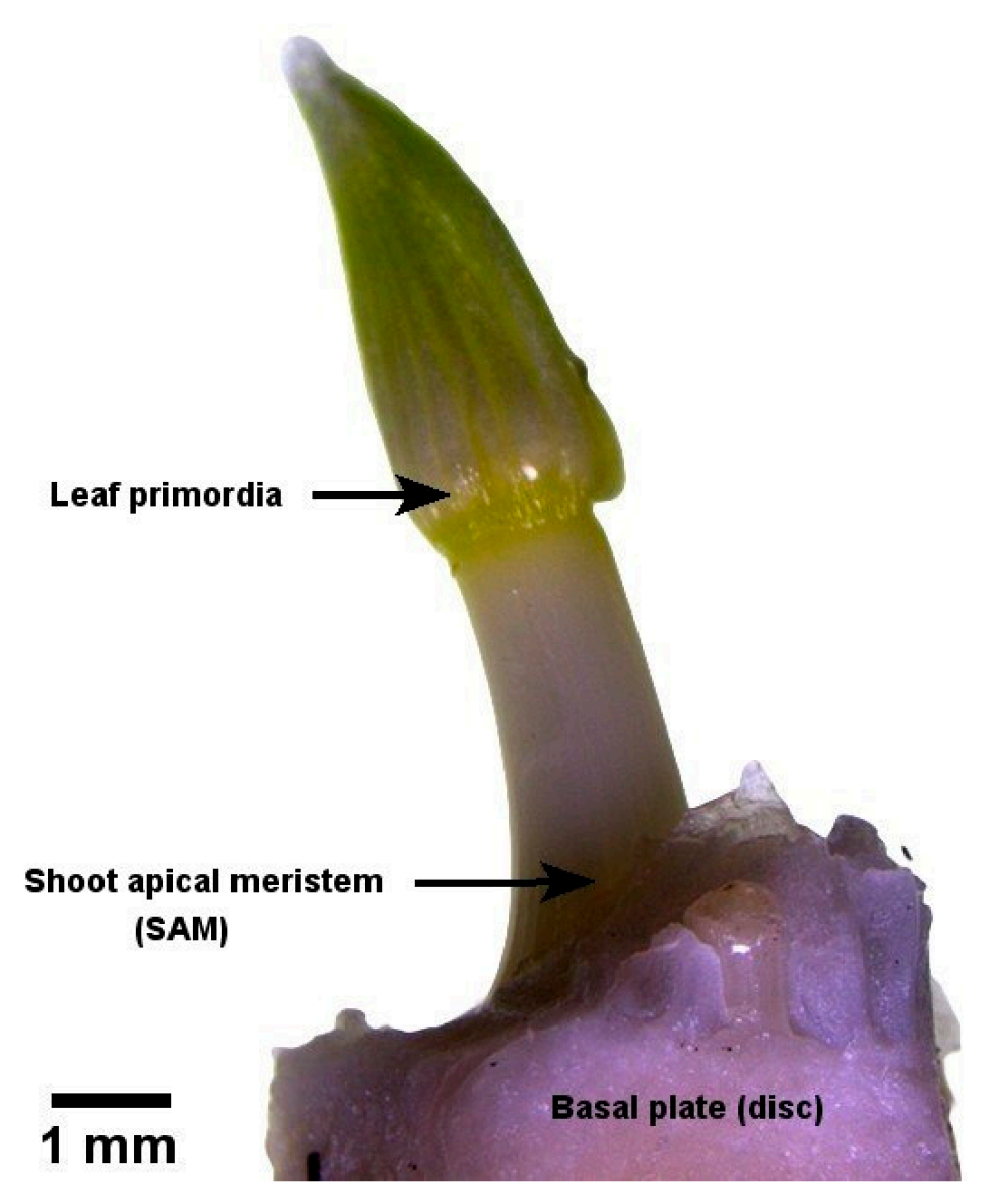
Shoot tip with leaf primordia used in shoot tip culture. Adapted from [56].

After 15 days in culture, plantlets regenerated from basal discs and shoot tips (6 to 10 mm long, including approx. 2 mm of basal disc) were excised (Figure 2). Shoot tip refers to the apical portion of the plant shoot, which includes the apical meristem (0.1 to 0.5 mm) and surrounding leaf primordia [43,71,72]. The excised shoot tips were cultured on M1 regeneration medium (Table S4) to induce plantlet formation. After one month of shoot tip culture, young leaves were collected from each plantlet for the post-culture viral detection. All plantlets were subcultured on M2 rooting medium (Table S4). After four weeks, when plantlets developed roots, they were transferred to M3 medium (Table S4) and then subcultured every 30 days. All plant material was kept at 25 ± 1 °C with a 16 h photoperiod under white light.

### 2.8. Acclimatization

When plants produced roots and bulbs, they were acclimatized. First, shallot plants were carefully removed from the 25 mL culture tubes, keeping the plants and their roots intact, and then rinsed with distilled water to remove the residual agar. They were then transferred to clay pots filled with autoclaved black soil in 1900 mL glass jars. Each jar was covered with plastic wrap and maintained at 25 °C with a 16 h photoperiod. Twice a week, plants were watered with sterile distilled water, and two holes were made in the plastic wrap. After one month, the plastic was completely removed. All plants received Hoagland fertilizer (Sigma-Aldrich, St. Louis, MO, USA) [73] every 15 days.

### 2.9. Data Collection and Statistical Analysis

The efficiency of the disinfection protocol was assessed by measuring the percentage of sterility (number of contamination-free plants/total number of plants) after 15 days of culture. We also determined the sprouting rate of basal discs after 15 days of culture, and the percentage of shoot tip explants that successfully regenerated into plants. The efficiency of the in vitro culture system was evaluated over 360 days based on sprouting response, plantlet development, rooting capacity, and bulb formation. Differences in sterility, plantlet growth, rooting, and bulb formation were analyzed using a Chi-squared test (χ^2^). One-way Analysis of Variance (ANOVA) was used to determine differences in the number of shoot tips excised per assay. A two-way ANOVA was applied to evaluate differences in rooting and bulb formation over time and across assays. All statistical analyses were performed using GraphPad Prism version 8.0.1 (GraphPad Software, San Diego, CA, USA), and statistical significance was established at *p* < 0.05. Virus elimination efficiency was assessed by determining whether the viruses were present or absent (PCR) following shoot tip culture.

## 3. Results

### 3.1. Viral Detection

#### 3.1.1. RNA Extraction

Total RNA extracted from the 99 shallot leaf samples (initial plant material) showed concentration averages that varied between 261.4 and 552.2 ng/µL (Table S5). The average RNA concentration from the 31 leaf samples of regenerated shallot plantlets ranged from 691.3 to 910.5 ng/µL (Table S6).

#### 3.1.2. Virus Detection Before Shoot Tip Culture

Of the 99 plants evaluated before in vitro culture, 87 were positive for SLV, 29 for IYSV, 27 for both viruses (co-infection), and 10 were negative for both viruses. Of the 27 samples coinfected, 72% belonged to Iq2 and 16% to Iq1 (Table 1). All samples analyzed tested negative for OYDV, ShVX, and LYSV viruses (Table 1). The identity of the viruses was confirmed by Sanger sequencing, with 82.28% to 83.87% for SLV and 100% for IYSV (Table S7). BLAST analyses of SLV sequences showed matches with sequences annotated as Garlic latent virus (GLV) in GenBank, which is recognized as a synonym of SLV in the literature [74].

### 3.2. Disinfection of Plant Material

The disinfection protocol for basal discs resulted in sterility rates of 47.8% for Assay 1, 32% for Assays 2 and 48% for Assay 3 (Table 2). Statistical analyses showed no significant differences in the sterility of the basal discs among the three assays (Chi-square test, *p =* 0.7899), indicating the reproducibility of the sterilization process.

### 3.3. Shoot Regeneration from Basal Discs

Sprouting of basal discs was 100% in all assays and controls after 15 days of culture.

### 3.4. Shoot Tip In Vitro Culture

**Figure 3:**
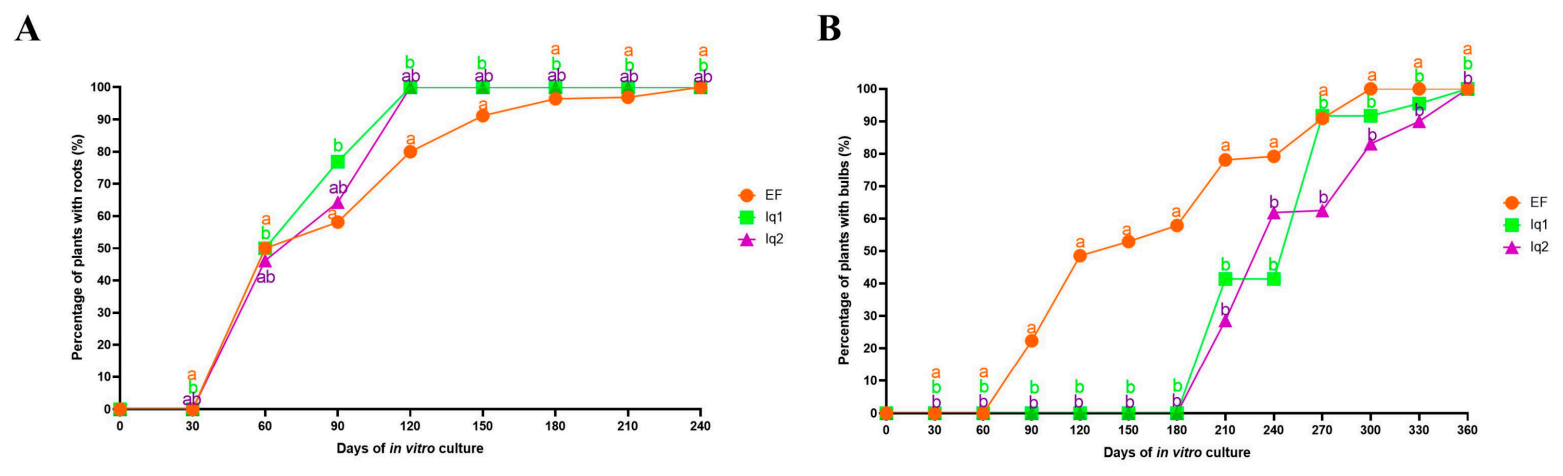
Percentage of rooting and bulb formation. Adapted from [56]. (**A**) Percentage of rooting throughout 250 days of in vitro culture. (**B**) Percentage of bulb formation throughout 360 days of in vitro culture. Data represent mean ± SD (*n* = X). Statistical significance was assessed using two-way ANOVA (rooting: days of culture, *p* < 0.0001; assay, *p* = 0.0419; bulb formation: days of culture, *p* = 0.0001; assay, *p* = 0.0004). Significant differences among assays are indicated by different letters within each group. Abbreviations: EF, Experimental Farm; Iq1, Iñaquito 1; Iq2, Iñaquito 2.

The mean of shoot tips obtained per basal disc was 6.5 for Assay 1, 5.5 for Assay 2, and 4.8 for Assay 3 (Figure S4). These differences were not statistically significant (one-way ANOVA, *p* = 0.2466). The percentage of plantlet development from shoot tips was highest in Assay 1 (88.7%) and lowest in Assay 2 (59.1%) (Table 3), with the difference being statistically significant (Chi-square test, *p* = 0.0084). In all assays, the regenerated plantlets developed into complete plants and successfully rooted (Figure 3A). Regarding bulb formation, it was faster in Assay 1, where plants began to form bulbs after 90 days of culture. In contrast, bulb development in Assays 2 and 3 was observed only after 210 days. By 300 days of culture, 100% of the plants in Assay 1 formed bulbs, whereas plants in Assays 2 and 3 required an additional 60 days to reach full bulb formation (Figure 3B). These differences were statistically significant (two-way ANOVA, *p* = 0.0004).

### 3.5. Detection of Plant Viruses After In Vitro Culture

After shoot tip culture, IYSV was not detected in any sample, indicating its successful elimination. However, SLV remained present in all shallot samples that had previously tested positive (Table 4, Table 5 and Table 6). Moreover, four samples that tested negative for SLV prior to in vitro culture were found to be positive afterward (Table 5 and Table 6).

## 4. Discussion

### 4.1. Effectiveness of Viral Detection

The results of this study revealed that our virus detection protocol using RT-PCR, with positive controls and a combination of specific and degenerate primers, was effective. The primers amplified the expected target region of the virus, enabling positive detection of SLV and IYSV in shallot samples. The analyzed shallots tested negative for OYDV, ShVX, and LYSV. OYDV has previously been reported in shallots and garlic in Ecuador [70,75], whereas LYSV and ShVX have been detected exclusively in garlic [76,77]. The absence of these viruses in our samples may be due to seasonal factors [78] or it may indicate that they are more common or confined to other *Allium* species, such as garlic, and have not yet spread to shallots. Future studies could consider using more sensitive techniques, such as nested RT-PCR [79] or digital PCR (ddPCR) [80], to detect very low levels of viral nucleic acids and confirm the presence and distribution of these viruses in shallot crops across Ecuador. Moreover, techniques such as multiplex RT-qPCR or high-throughput sequencing (including RNA-seq) [81,82,83,84] could be employed for quantitative detection and characterization of known and unknown viral variants.

The presence of SLV in 87.9% of the analyzed shallot plants indicates that this virus is widely distributed within the sampled population. IYSV was present in 29.3% of shallot samples, with 27.3% showing co-infection with both viruses. SLV, a member of the *Carlavirus* genus and *Betaflexiviridae* family, is easily transmitted through sap and by aphid vectors such as *Myzus ascalonicus* and possibly *Aphis fabae* in a non-persistent manner [7]. Its presence is well documented, having been detected in shallots [70] and garlic (*Allium sativum*) [77] in Ecuador, with reported SLV detection rates of 100% in shallots and approximately 88.7% in garlic. This virus has also been reported in other American countries such as Brazil, Mexico, and Argentina [85,86,87], as well as in Europe and Asia, including Hungary, India, and China [60,88,89].

Interestingly, up to 10.1% of samples tested negative for SLV, suggesting that although SLV is widespread in shallot plants, it is not always present in all samples. These negative cases were detected in the Iq1 and Iq2 markets, with Iq2 also showing a higher incidence of IYSV infection. This variation in viral presence could be influenced by crop management practices, including water management, crop rotation, fertilization, and pest and disease control measures, such as chemical pesticides [90,91,92]. Other studies have reported diverse viral profiles in shallots, including cases in which SLV was less dominant than other viruses. For example, a study from Indonesia reported SLV infection rates ranging from 60% to 78% [93]. Another study in France reported some shallot samples negative for SLV but positive for ShVX and for newly identified carlaviruses and potyviruses [54]. Collectively, these findings suggest that shallots are susceptible to diverse viral infections and that SLV is not consistently present in all populations.

The other virus detected in our samples was IYSV, which belongs to the *Tospovirus* genus and *Bunyaviridae* family and represents an emerging threat to onion bulb and seed production [94]. Symptoms produced by IYSV include diamond-shaped lesions of 1–5 cm limited by a chlorotic zone resulting from chloroplast malformation [95,96,97,98]. These symptoms were observed on some shallot leaves from OM, Iq1, and Iq2, but not in EF. Subsequent molecular analyses revealed a correlation between the observed symptoms and the presence of the viruses. The differences in IYSV occurrence among sample sources may be associated with variations in agronomic practices, the origin of planting material, vector pressure, and local environmental conditions that influence virus transmission and disease development [99]. However, these factors were not evaluated in the present study and should be investigated in future work to elucidate their role in IYSV epidemiology. Although IYSV had not been previously reported in shallots or garlic in Ecuador, it has been detected in onions [100]. To our knowledge, this is the first report of IYSV infecting shallots in Ecuador. Given that IYSV infects a wide range of *Allium* species worldwide—including onion, shallot, leek, garlic, and chive—in Europe (the Netherlands, Germany, Spain, France, and Austria), the Americas (the United States, Mexico, Peru, Brazil, and Uruguay), Africa (South Africa), Asia (Japan and India), Oceania (Australia), and the Mediterranean region [7,53,101,102,103,104,105,106,107,108,109,110,111,112,113], these findings are particularly noteworthy. Given the agricultural importance of *Allium* crops in Ecuador, implementation of early preventive measures against IYSV could be highly beneficial in the long term.

The transmission of IYSV is due to vectors and virus reservoirs in plants [114]. The main vector, *Thrips tabaci* Lindeman, commonly known as “onion trips”, transmits the virus in a persistent and propagative manner [99,115]. The detection of IYSV in shallots and onions in Ecuador probably indicates the presence of this vector. A recent study confirmed the presence of *Thrips palmi* and *Thrips tabaci* in Ecuador [116]. Although there is limited evidence of crops being affected by *T. tabaci* in the country, this warrants further investigation, as it is the main vector of IYSV. Moreover, this virus can infect other *Allium* species, such as garlic and onion, which are also widely cultivated in Ecuador [76,100,117]. Infection with IYSV also makes the plant more vulnerable to environmental stresses such as drought, high temperatures, and excessive irrigation [7,115]. Moreover, some secondary pathogens, such as *Alternaria spp*. and *Stemphylum spp*., can colonize those necrotic viral lesions [101], complicating the diagnosis. Therefore, early IYSV detection is critical for implementing sustainable control and prevention methods to limit viral spread and reduce potential economic crop losses in the future. In addition, accurate identification of the vector and study of its biology could provide valuable insights to help growers manage this disease, particularly in countries like Ecuador, where *Allium* crops are commonly used and cultivated.

Several viruses can simultaneously infect shallots, resulting in mixed infections within a single plant [54]. Such coinfections were observed in samples from OM, Iq1, and Iq2. Notably, infections of IYSV with *Carlavirus* and *Allexivirus* have been documented, and their concurrent presence often produces a synergistic effect that intensifies symptoms and causes severe yield losses [118]. This situation poses particular challenges for farmers in rural areas, who may lack the necessary information and resources to manage these complex infections effectively. In addition, virus monitoring in shallots and other *Allium* crops in the country remains limited and is less well documented than in other countries. These findings underscore the importance of government agricultural agencies establishing routine screening programmes not only for the viruses identified in this study but for additional viruses that may threaten crop production.

### 4.2. In Vitro Shoot Tip Culture of Shallot

Our standardized disinfection protocol achieved sterility in 32% to 48% of shallot basal disc assays. Although these percentages were lower than expected, they provided enough sterile plant material for subsequent in vitro assays. Fungal pathogens are the most common biotic constraints affecting shallots, especially *Fusarium oxysporum*, *Spodoptera exigua,* and *Alternaria porri*, each causing a distinct disease in the plant [119]. Consequently, selecting an effective fungicide to obtain sterile basal discs was essential. We used Mertect, which contains thiabendazole, a compound known for its effectiveness and broad-spectrum of activity against various pathogenic fungi [120]. Benomyl is often recommended and has been widely reported to inhibit most fungal taxa [121,122], with 100% sterility in shallots [70]; however, it is banned in Ecuador [123] due to its teratogenic and toxic effects [124].

In all assays, 100% basal disc sprouting was achieved following the sterilization protocol. This result is emphasized because, unlike previous studies by Vega et al. [47] and Ramírez [70], we did not use a drying or cooling treatment for the shallot bulbs [125,126]; all the bulbs were fresh. Our results showed that all discs sprouted after only 15 days of culture, demonstrating the efficiency of our protocol.

More than 59.1% of shoot tips developed into plantlets, 100% of shallots developed roots, and over 83.1% of plantlets produced bulbs after up to 300 days of culture. The differences observed in rooting and bulb formation between assays throughout 360 days (Figure 3) are more likely attributable to variations in environmental conditions and handling of the source plant material rather than to differences in the in vitro culture conditions, which were identical across all assays. Rapid root system development is a key strategy for plant survival and growth [127], and these regeneration rates are notably higher than those reported in previous studies by Vega et al. [47] and Ramírez [70], which achieved only 30% to 40% and 68%, respectively. In this study, adding ancymidol (an inhibitor of gibberellin biosynthesis) to the M3 medium significantly increased the percentage of bulb formation. Ancymidol promotes bulbing and rhizogenesis by reducing sucrose levels at the leaf bases and increasing the contents of glucose, fructan, and fructose [128]. The observed bulb formation rate surpassed the 77% previously reported [47], underscoring the effectiveness of this approach.

All rooted and bulb-forming plants were successfully acclimatized, representing a clear improvement over previous studies in which acclimatization was either not achieved [70] or only partially successful [47]. Although all shallot’s developmental stages were successfully achieved, we observed that shoot tips derived from mother plants infected only with SLV appeared to develop and grow faster than those derived from plants infected with both viruses, although this was not quantitatively measured. In future studies, it would be interesting to include quantitative comparisons among plants with different virus infection statuses or virus-free controls to determine whether initial virus infection influences the success of regeneration, rooting, or bulb formation. Further research is needed to clarify all factors influencing shallot development, particularly bulb formation, and to enhance the production and selection of high-quality plants for in vitro propagation. Overall, shoot tip culture proved effective, yielding high regeneration rates and efficient bulb formation, making it a promising tool for in vitro research on shallots.

To explore the virus elimination potential of shoot tip culture, we evaluated its effectiveness against both viruses. The shoot tip culture effectively removed IYSV, resulting in 100% virus-free plants, regardless of the source (Iq1 or Iq2). Since 1999, IYSV has been included in the European and Mediterranean Plant Protection Organization (EPPO) alert list due to its potential hazard to onion crops [113,129]. It is an industry concern due to the limited availability of effective treatment options to control the virus and its vector [130]. To date, no chemical or biological methods have been shown to eliminate IYSV from infected plants. IYSV management relies on preventive strategies, including the destruction of infected plants and vector control [94,131,132,133]. The complete elimination of IYSV following shoot tip culture may be explained by the virus’s limited ability to invade the youngest meristematic tissues. These tissues lack vascular connections, undergo rapid cell division, and exhibit high metabolic activity [134,135], conditions that can restrict viral replication and movement. In addition, physiological and metabolic changes associated with shoot tip culture may activate antiviral defence responses in plants, as reported for other virus elimination techniques, such as thermotherapy [134]. Antiviral immunity mediated by RNA interference and salicylic acid may also play an important role in excluding several RNA viruses from stem cells [136]. Together, these factors may reduce viral loads and ultimately lead to the elimination of IYSV from the plant. Our study demonstrates the effectiveness of shoot tip culture for eliminating IYSV and highlights its potential application as a preventive plant protection strategy. IYSV-free shallots can be integrated into production systems as clean planting material before field establishment, reducing the risk of virus introduction and subsequent spread and contributing to the long-term sustainability of shallot production.

Coinfections are more challenging to eliminate than infections caused by a single virus [137,138], and virus–host interactions likely affect eradication success [139,140]. However, the precise reasons behind this remain unclear due to its complexity [139,141]. SLV is generally asymptomatic and, on its own, is not considered to cause significant economic losses in shallot crops. However, coinfections with other viruses, such as IYSV, may intensify the disease symptoms and reduce plant vigour and yield [7,54]. In this case, although SLV could not be eliminated, eliminating IYSV may benefit co-infected plants by reducing overall viral load and improving plant health, thereby allowing for continued growth and development.

A factor that may contribute to SLV persistence is explant size, which is critical for successful virus eradication, particularly for latent viruses [140]. In our study, we selected shoot tips measuring 6–10 mm, as smaller explants failed to regenerate in pilot experiments. While shoot tips contain the meristem, they also include leaf primordia tissue, and some viruses are known to migrate through the vascular elements [142]. Meristem culture using explants of 0.5–0.8 mm has produced 25–50% virus-free garlic and shallot plants for SLV and OYDV [143], while other studies report higher eradication rates even with smaller meristem sizes [45,139,144]. For example, Verbeek et al. [45] obtained 91 to 100% OYDV-G-free garlic plants using meristems of 0.21 to 0.33 mm, while cultures of 0.21 mm meristems produced 62–65% virus-free garlic plants for OYDV, LYSV, and GCLV [144]. In shallots, Wang et al. [139] found that 0.5 mm meristem culture resulted in only 10% OYDV and 15% SLV-free plants. Conversely, larger explants such as shoot tips produce plants with viral elimination rates of 96.2% for SLV and OYDV by the third generation [52]. Generally, smaller explants improve virus elimination but reduce survival and regeneration rates [43,145,146]. Since one of the main objectives of in vitro culture is to generate new plants, selecting an appropriate explant size requires balancing these factors. The size of shoot tips used in our study allowed for both a high rate of regeneration and viral elimination of more symptomatic viruses.

SLV is a complex virus that is difficult to fully eliminate with a single treatment. Bradamante et al. [147] suggest that some viruses can also evade the antiviral barriers of the meristem. Supporting this, Magyar-Tábori [148] found that only 20– 27% of the 0.5 mm shallot meristematic region was free of SLV, suggesting that combined methods may be necessary for complete or higher elimination. This study serves as a preliminary screening before testing other or integrated treatments. Therefore, future efforts should focus on integrated approaches that combine methods such as thermotherapy, chemotherapy, and shoot tip culture to achieve higher eradication rates for SLV [36,139].

Unexpectedly, four shallot samples from assays 2 and 3 tested negative for SLV before in vitro culture and positive after shoot tip culture. This result might be explained by low initial viral loads below RT-PCR detection thresholds. Although the limit of detection (LOD) was not experimentally measured, conventional RT-PCR assays have finite detection thresholds, and samples containing low viral copy numbers may fail to produce a visible amplification product [149,150]. In addition, plant viruses are often unevenly distributed within host tissues [148,151]. SLV levels appeared to fluctuate across plant developmental stages [152]. Velásquez-Valle [153] also showed that virus concentrations tend to increase after an ineffective eradication treatment, suggesting that the virus may have replicated and accumulated during shoot tip culture, making it detectable later. Studies on SLV replication mechanisms are limited, but in a few cases, latent virus titers may vary under certain conditions, such as those induced by environmental stress (produced by the in vitro culture process itself) or during specific developmental stages of the host plant [154,155,156].

Despite the insights provided by this study, there are certain limitations that should be acknowledged. Although shoot tip culture successfully eliminated IYSV, its effectiveness against SLV was limited. In addition, viral detection was based on RT-PCR rather than high-throughput sequencing, and viral loads were not quantified. Plants were monitored for approximately 360 days; however, longer-term evaluation after acclimatization would provide additional evidence for the long-term stability of virus elimination. Future studies incorporating high-throughput sequencing, viral quantification, and extended post-acclimatization monitoring would further strengthen these findings.

## 5. Conclusions

This study reports an efficient protocol for the molecular detection of two of the most common viruses in shallot crops, SLV and IYSV. To our knowledge, this is the first report of IYSV in shallots in Ecuador [56]. This detection may support the implementation of control, monitoring, and prevention measures to limit the spread of the virus and to reduce the economic and crop losses in shallots and other *Allium* crops. The presence of the virus implies the presence of its vector [99,117]. Now that *Thrips tabaci* has been reported in Ecuador [116], understanding its life cycle and interactions with IYSV could help growers control the disease. The results of our in vitro shoot tip culture proved effective in eliminating IYSV, which in other countries has caused significant economic losses by impacting crop quality and yield, with no chemical or biological methods available to eliminate the virus in the plant [94]. Although our study focused on the detection of five viruses, other viruses may be present in shallots, making third-generation sequencing a worthwhile approach to understanding the complete shallot virome. The in vitro culture was efficient, resulting in a high percentage of plant development. Therefore, in vitro shoot tip culture could be a valuable tool for future research and an effective method to eradicate viruses in shallots. Performing multiple rounds of shoot tip culture could further ensure the production of truly virus-free plants. Good-quality plants (free of IYSV and effectively acclimatized) obtained in this study were distributed to local farmers, providing them with high-quality planting material for field propagation. Research of this kind not only advances scientific knowledge but also provides valuable insights for improving agricultural practices.

## Figures and Tables

**Table 1:** Viral detection in shallot plants before in vitro culture. Adapted from [56].

Source	Plants Analyzed (*n*)	Single Infection		Coinfection	SLV and IYSV Negative *n* (%)	Total SLV Positive *n* (%)	Total IYSV Positive *n* (%)
		SLV *n* (%)	IYSV *n* (%)	SLV + IYSV *n* (%)			
EF	23	23 (100)	0 (0.0)	0 (0.00)	0 (0.0)	23 (100.0)	0 (0.0)
OM	26	21 (80.8)	0(0.0)	5 (19.2)	0 (0.0)	26 (100.0)	5 (19.2)
Iq1	25	10 (40.0)	2(8.0)	4 (16.0)	9 (36.0)	14 (56.0)	6 (24.0)
Iq2	25	6 (24.0)	0(0.0)	18 (72.0)	1 (4.0)	24 (96.0)	18 (72.0)
Total	99	60 (60.6)	2 (2.0)	27 (27.3)	10 (10.1)	87 (87.9)	29 (29.3)

**Table 2:** Sterility and sprouting rates of basal discs of shallots after 15 days of in vitro culture. Adapted from [56].

Assay	Source	Sterility ^1^ (%)	Sprouting (%)
**1**	EF	11/23 (47.8)	23/23 (100)
**2**	Iq1	8/25 (32)	25/25 (100)
**3**	Iq2	12/25 (48)	25/25 (100)
**Mean**		31/73 (42.5)	73/73 (100)

^1^ Statistical significance was assessed using Chi-square (sterility: *p* = 0.7899).

**Table 3:** Percentages of plantlet development, rooting, and bulb formation.

Assay	Source	Shoot Tips (*n*)	Plant Development (%)	Rooting (%)	Bulb Formation at 300 Days (%)	Bulb Formation ^b^ at 360 Days (%)
**1**	EF	71	63/71 (88.7)	74/74 (100) ^a^	74/74 (100)	74/74 (100)
**2**	Iq1	44	26/44 (59.1)	36/36 (100)	33/36 (91.7)	36/36 (100)
**3**	Iq2	58	45/58 (77.6)	65/65 (100) ^a^	54/65 (83.1)	65/65 (100)

^a^ Plantlet number increased after subculture; subsequent percentages were calculated based on total plantlets. ^b^ Bulb formation data up to 360 days of culture. Statistical significance was assessed using Chi-square (plant development: *p* = 0.0084; bulb formation by 300 days: *p* = 0.0092).

**Table 4:** Virus detection results in shallots from the experimental farm (Assay 1), before and after in vitro culture. Adapted from [56].

Samples	SLV Before	SLV After	IYSV Before	IYSV After
1	+	+	−	−
2	+	+	−	−
3	+	+	−	−
4	+	+	−	−
5	+	+	−	−
6	+	+	−	−
7	+	+	−	−
8	+	+	−	−
9	+	+	−	−
10	+	+	−	−
11	+	+	−	−

Abbreviations: SLV, Shallot Latent Virus; IYSV, Iris Yellow Spot Virus.

**Table 5:** Virus detection results in shallots from market Iq1 (Assay 2), before and after in vitro culture. Adapted from [56].

Samples	SLV Before	SLV After	IYSV Before	IYSV After
1	+	+	+	−
2	−	+ ^1^	−	−
3	+	+	−	−
4	−	+ ^1^	+	−
5	+	+	−	−
6	−	+ ^1^	−	−
7	+	+	+	−
8	+	+	−	−

Abbreviations: SLV, Shallot Latent Virus; IYSV, Iris Yellow Spot Virus. ^1^ Samples that tested negative before and positive after the disinfection protocol.

**Table 6:** Virus detection results in shallots from market Iq2 (Assay 3), before and after in vitro culture. Adapted from [56].

Samples	SLV Before	SLV After	IYSV Before	IYSV After
1	+	+	−	−
2	+	+	+	−
3	+	+	+	−
4	+	+	+	−
5	+	+	+	−
6	+	+	+	−
7	+	−	-	−
8	+	+	+	−
9	−	+ ^1^	−	−
10	+	+	+	−
11	+	+	+	−
12	+	+	+	−

Abbreviations: SLV, Shallot Latent Virus; IYSV, Iris Yellow Spot Virus. ^1^ Samples that tested negative before and positive after the disinfection protocol.

## Data Availability

All original contributions are included in the article and Supplementary Materials; further inquiries should be directed to the corresponding authors. The GenBank accession numbers of the sequences generated in this study can be found in Supplementary Table S7.

## Supplementary Materials

The following supporting information can be downloaded at: https://www.mdpi.com/article/10.3390/pathogens15080799/s1, Table S1: Primers tested in a pilot study; Table S2: Primers and target genes used in the study; Table S3: Disinfection protocol for shallot bulbs; Table S4: Media composition obtained from Vega et al. [47]; Table S5: Quantification of total RNA extracted from shallot leaves and absorbance index before in vitro culture; Table S6: Quantification of total RNA extracted from shallot leaves and absorbance index after in vitro culture. Table S7: Sanger sequencing results for RT-PCR amplicons of shallot viruses. Figure S1: Amplification of PCR products of positive controls with designed and literature-based primers. (A) PCR products of SLVLIT with positive controls. (B) PCR products of SLVDIS with positive controls. (C) PCR products of OYDVLIT with positive controls. (D) PCR products of OYDVDIS with positive controls. (E). PCR products of ShVXDIS1 with positive controls. (F) PCR products of ShVXDIS2 with positive controls. (G) PCR products of IYSVLIT with positive controls. (H) PCR products of IYSVDIS with positive controls. (I) PCR products from LYSVLIT with positive controls. (J) PCR products of LYSVDIS with positive controls. The lack of a band implies the absence of the virus. Abbreviations: Ladder: ladder, C−: negative control, C+: positive control; Figure S2: Pilot evaluation of PCR products of shallot samples with designed and literature-based primers. (A) PCR products of SLV with literature primers. (B) PCR products of SLV with designed primers. (C,D) PCR products of OYDV with literature primers. (E,F) PCR products of ShVX with designed primers. (G,H) PCR products of IYSV with designed primers. (I,J) PCR products of LYSV with designed primers. The lack of a band implies the absence of the virus. Abbreviations: L: ladder, C+: positive control; Figure S3: Scheme of shallot shoot regeneration from shoot tips. (A) Washing bulbs with tap water. (B) Washing bulbs with Mertect fungicide (15 min). (C) Disinfection protocol with ET-OH 70%, NaClO 4.5%, and Tween 20 inside the laminar flow chamber. (D) Culture of basal discs (M1 medium). (E) Shoot tip culture (M1 medium). (F) Rooting (M2 medium). (G) Bulb formation (M3 medium). (H) Acclimatization of shallots; Figure S4: The mean number of shoots tips extracted per basal disc under different assays. Data represent mean ± SD (*n* = X). Statistical significance was assessed by one-way ANOVA (*p* = 0.2466). Abbreviation: EF, Experimental Farm; Iq1, Iñaquito 1; Iq2, Iñaquito 2.
