# Supplementary material for: Unraveling Shallot Viral Diversity: PCR Detection and In Vitro Culture
Source: Pathogens. 2026 Jul 28;15(8):799. doi: 10.3390/pathogens15080799 (PMC13516031; doi:10.3390/pathogens15080799)
Supplement: Supplementary file 1 [file pathogens-15-00799-s001.zip › Suplementary Material-ID 4355948.pdf]

Supplementary Material

Journal name: Pathogens

Title:

Unraveling Shallot Viral Diversity: PCR Detection and *In Vitro* Culture

Author names:

Kelly Zúñiga-Vera[1,2], Martina Albuja-Quintana[1], Diana Calderón[1], Miguel Orellana[1], Carlos Ruales[3], and Maria de Lourdes Torres[1*]

Author affiliations:

[1] Laboratorio de Biotecnología Vegetal, Colegio de Ciencias Biológicas y Ambientales, Universidad San Francisco de Quito, Quito 170901, Ecuador.
[2] Instituto de Microbiología, Colegio de Ciencias Biológicas y Ambientales, Universidad San Francisco de Quito, Quito 170901, Ecuador.
[3] Laboratorio de Biotecnología Agrícola y de Alimentos, Colegio de Ciencias E Ingenierías- Ing. Agronomía, Universidad San Francisco de Quito, Quito 170901, Ecuador.

***Corresponding author**: ltorres@usfq.edu.ec

Table S1. Primers tested in a pilot study.

Source	Virus[1]	Oligo Name	Target Region		Primer (5' - 3')	Expected size
**Majumder et al. [1]**	SLV	SLVLIT	Coat protein	Fwd	GTGGTNTGGAATTAC	308 bp
				Rev	CAACATCGATTYTCTC	
**Designed**		SLVDIS	Coat protein	Fwd	cGgAAcTAcGCaCCtGARAG	215 bp
				Rev	ACcACCATgTCcCTaGTYaC	
**Mahmoud et al. [2]**	OYDV	OYDVLIT	Coat protein	Fwd	CGAAGCAAATTGCCAAGCAG	625 bp
				Rev	CGATTAGCTGCCCCTCTAAC	
**Designed**		OYDVDIS	Coat protein	Fwd	ACtATGATGGATGGYgAYGA	170 bp
				Rev	TTtCGCTGAAGTCCaTACCG	
**Designed**	ShVX	ShVXDIS1	Replicase	Fwd	CcGtcGTGAATAGCCAAagG	160 bp
				Rev	GGtTCgAGgTtTGStGARGA	
**Designed**		ShVXDIS2	Replicase	Fwd	ACTCTTACACYGGCTACTCG	221 bp
				Rev	tATGCCaAAtgTtCCgGAGC	
**Leach et al. [3]**	IYSV	IYSVLIT	Nucleocapsid	Fwd	ACTCACCAATGTCTTCAAC	402 bp
				Rev	GGCTTCCTCTGGTRAGTGC	
**Designed**		IYSVDIS	Nucleocapsid	Fwd	TCCTTTGCTGCCATGACTCT	189 bp
				Rev	TGMAACCACAGCCAGGATCA	
**Katis et al. [4]**	LYSV	LYSVLIT	Coat protein	Fwd	AAGAACACCAGTTAGAGCGCG	126 bp
				Rev	TGCCTCTCCGTGTCCTCATC	
**Designed**		LYSVDIS	Coat protein	Fwd	ATGGCgCAtTTYTCaGCAC	191 bp
				Rev	GCTGCTTTCATTTGTGCRtG	

[1] Abbreviation: SLV, Shallot Latent Virus; OYDV, Onion Yellow Dwarf Virus; ShVX, Shallot Virus X; IYSV, Iris Yellow Spot Virus; and LYSV, Leek Yellow Stripe Virus.

Table S2. Primers and target genes used in the study.

Source	Virus[1]	Oligo Name	Target region		Primer (5' - 3')	Expected size
Majumder et al. [1]	SLV	SLVLIT	Coat protein	Fwd	GTGGTNTGGAATTAC	308 bp
				Rev	CAACATCGATTYTCTC	
Mahmoud et al. [2]	OYDV	OYDVLIT	Coat protein	Fwd	CGAAGCAAATTGCCAAGCAG	625 bp
				Rev	CGATTAGCTGCCCCTCTAAC	
Designed	ShVX	ShVXDIS	Replicase	Fwd	CCGTCGTGAATAGCCAAAGG	160 bp
				Rev	GGTTCGAGGTTTGSTGARGA	
Designed	IYSV	IYSVDIS	Nucleocapsid	Fwd	TCCTTTGCTGCCATGACTCT	189 bp
				Rev	TGMAACCACAGCCAGGATCA	
Designed	LYSV	LYSVDIS	Coat protein	Fwd	ATGGCGCATTTYTCAGCAC	191 bp
				Rev	GCTGCTTTCATTTGTGCRTG	

[1] Abbreviation: SLV, Shallot Latent Virus; OYDV, Onion Yellow Dwarf Virus; ShVX, Shallot Virus X; IYSV, Iris Yellow Spot Virus; and LYSV, Leek Yellow Stripe Virus.

Table S3. Disinfection protocol for shallot bulbs.

Washing time with Mertect fungicide	Chemical disinfection protocol	Initial culture media
3.5 g/L for 15 min.	Et-OH (70%) for 10 min.<br>NaClO (4.5%) + Tween-20 for 35 min.<br>Washes with sterile dH2O	M1: MS + NAA + BAP

Table S4. Media composition obtained from Vega et al. [5].

Medium	Composition
Murashige & Skoog (MS)	100ml Major Elements, 10ml Minor Elements, 50ml Organic Elements, 10ml FeDA, sucrose 30 g/L, and agar 8 g/L.
M1	MS medium, NAA (1.1 μM), BAP (8.9 μM), sucrose 30 g/L, and agar 8 g/L.
M2	MS medium.
M3	MS medium, NaPO4 300 mg/L, ancymidol 10 μM, sucrose 50 g/L, and agar 8 g/L.

Table S5. Quantification of total RNA extracted from shallot leaves and absorbance index before *in vitro* culture.

Sample	Experimental farm			Organic market			Iñaquito market 1			Iñaquito market 2		
	**ng/µL**	**260/280**	**260/230**	**ng/µL**	**260/280**	**260/230**	**ng/µL**	**260/280**	**260/230**	**ng/µL**	**260/280**	**260/230**
1	764,00	2,01	2,43	616,00	1,90	0,83	590,00	2,01	1,60	636,00	1,72	0,40
2	554,00	2,03	2,18	448,00	1,78	1,07	588,00	1,95	0,91	196,00	1,89	1,34
3	386,00	1,97	2,31	748,00	1,98	1,26	626,00	1,85	0,65	214,00	1,95	1,39
4	686,00	1,97	2,01	608,00	1,99	1,77	101,00	1,91	0,41	364,00	1,94	0,96
5	768,00	1,94	2,36	798,00	1,84	0,66	732,00	2,01	1,48	454,00	2,03	1,83
6	324,00	1,96	1,25	866,00	2,01	1,24	276,00	1,95	0,56	121,00	1,82	0,42
7	800,00	2,04	2,26	892,00	2,05	1,32	474,00	2,02	1,39	149,00	1,87	0,75
8	738,00	2,04	2,36	546,00	1,94	0,99	1080,00	2,06	1,14	110,00	1,76	0,31
9	430,00	1,98	1,36	656,00	1,94	0,99	286,00	1,88	0,78	199,00	1,95	1,94
10	656,00	2,01	2,29	200,00	1,92	1,98	382,00	1,98	1,50	486,00	2,01	2,03
11	328,00	1,95	2,21	324,00	1,98	2,21	226,00	1,99	1,56	234,00	2,01	1,21
12	404,00	1,89	1,76	264,00	1,98	2,03	76,00	1,77	0,64	149,00	2,01	1,23
13	994,00	2,05	2,34	172,00	1,91	1,02	1200,00	2,01	2,15	234,00	1,98	2,02
14	426,00	2,08	2,33	904,00	2,02	2,44	62,20	1,97	1,03	462,00	1,97	1,74
15	426,00	1,97	2,27	272,00	1,93	1,85	177,00	1,89	1,43	264,00	1,96	1,78
16	526,00	1,92	2,12	234,00	1,98	1,90	127,00	1,89	0,35	338,00	1,94	2,06
17	320,00	1,91	1,82	164,00	1,98	1,53	814,00	1,94	1,43	220,00	1,99	0,83
18	808,00	2,01	2,29	41.6	1,83	0,52	660,00	2,05	2,37	80,00	2,88	0,89
19	438,00	1,93	1,82	324,00	1,91	0,47	578,00	1,87	1,53	302,00	1,99	1,23
20	740,00	1,94	2,44	440,00	1,97	1,10	602,00	1,96	2,21	-*	1,97	0,81
21	466,00	1,91	1,76	630,00	2,03	1,35	812,00	2,01	2,33	256,00	1,85	0,58
22	280,00	1,94	1,50	410,00	1,98	1,10	412,00	1,99	2,36	80,60	3,23	0,71
23	438,00	1,97	2,03	1020,00	1,97	1,97	970,00	1,98	2,32	318,00	1,87	1,14
24	-	-	-	514,00	1,71	1,09	302,00	1,91	0,64	185,00	1,76	0,24
25	-	-	-	562,00	1,79	1,33	192,00	1,84	1,07	222,00	1,82	0,93
26	-	-	-	185,00	1,74	0,86	-	-	-	-	-	-
**Mean**	**552,20**	**1,98**	**2,07**	**508,20**	**1,93**	**1,34**	**493,80**	**1,95**	**1,35**	**261,40**	**2,01**	**1,15**
**S**	**199,20**	**0,05**	**0,35**	**254,10**	**0,09**	**0,53**	**320,90**	**0,07**	**0,65**	**138,90**	**0,33**	**0,58**
**Min**	**280,00**	**1,89**	**1,25**	**164,00**	**1,71**	**0,47**	**62,20**	**1,77**	**0,35**	**80,00**	**1,72**	**0,24**
**Max**	**994,00**	**2,08**	**2,44**	**1020,00**	**2,05**	**2,44**	**1200,00**	**2,06**	**2,37**	**636,00**	**3,23**	**2,06**

Table S6. Quantification of total RNA extracted from shallot leaves and absorbance index after *in vitro* culture.

**Sample**	**Assay 1**			**Assay 2**			**Assay 3**		
	**ng/µL**	**260/280**	**260/230**	**ng/µL**	**260/280**	**260/230**	**ng/µL**	**260/280**	**260/230**
1	798	1,99	2,38	650	1,97	1,96	638	2,08	1,78
2	506	1,97	2,2	1180	2,00	2,3	426	2,05	2,39
3	764	2,03	2,33	802	1,97	1,92	832	2,07	1,77
4	826	1,03	2,46	1080	2,08	2,38	328	2,07	2,37
5	750	2,03	2,31	1060	2,08	2,43	1120	1,98	1,84
6	1100	2,01	2,4	1100	2,08	2,2	248	1,61	0,29
7	1020	2,00	2,00	802	2,02	2,22	-*	1,55	0,14
8	1100	2,02	2,16	610	2,00	2,21	1140	2,02	2,02
9	364	2,04	2,47	-	-	-	1080	1,97	2,38
10	258	2,06	2,4	-	-	-	616	1,78	1,42
11	118	2,04	2,35	-	-	-	712	2,04	2,3
12	-	-	-	-	-	-	808	2,04	2,22
**Mean**	**691,30**	**1,93**	**2,32**	**910,50**	**2,03**	**2,20**	**722,50**	**1,94**	**1,74**
**SD**	**337,40**	**0,30**	**0,14**	**220,80**	**0,05**	**0,18**	**310,90**	**0,19**	**0,78**
**Min**	**118,00**	**2,06**	**2,47**	**610,00**	**1,97**	**1,92**	**248,00**	**1,55**	**0,14**
**Max**	**1100,00**	**1,03**	**2,00**	**1180,00**	**2,08**	**2,43**	**1140,00**	**2,08**	**2,39**

Table S7. Sanger sequencing results for RT-PCR amplicons of shallot viruses.

No.	Sequence ID	Host	Isolation Source	Virus	Target gene	Sequencing method	GenBank accession number	Closest GenBank isolate	% Identity
1	SHAL-SLV-03	*Allium cepa* var. *Aggregatum*	Shallot leaf / Leaf tissue	*Shallot latent virus (SLV)	Coat protein	Sanger sequencing	PZ695405	*Garlic latent virus isolate G35, complete genome (MN059211.1)	83.87%
2	SHAL-SLV-04	*Allium cepa* var. *Aggregatum*	Shallot leaf / Leaf tissue	*Shallot latent virus (SLV)	Coat protein	Sanger sequencing	PZ695406	*Garlic latent virus isolate G35, complete genome (MN059211.1)	83.39%
3	SHAL-SLV-05	*Allium cepa* var. *Aggregatum*	Shallot leaf / Leaf tissue	*Shallot latent virus (SLV)	Coat protein	Sanger sequencing	PZ695407	*Garlic latent virus isolate G35, complete genome (MN059211.1)	82.28%
4	SHAL-IYSV-92	*Allium cepa* var. *Aggregatum*	Shallot leaf / Leaf tissue	Iris yellow spot virus (IYSV)	Nucleocapsid	Sanger sequencing	PZ715995	Iris yellow spot virus isolate IYSV-Lisianthus nucleocapsid-like (N) gene, partial sequence (HM035070.1)	100%
5	SHAL-IYSV-97	*Allium cepa* var. *Aggregatum*	Shallot leaf / Leaf tissue	Iris yellow spot virus (IYSV)	Nucleocapsid	Sanger sequencing	PZ715996	Iris yellow spot virus isolate IYSV-Lisianthus nucleocapsid-like (N) gene, partial sequence (HM035070.1)	100%
6	SHAL-IYSV-99	*Allium cepa* var. *Aggregatum*	Shallot leaf / Leaf tissue	Iris yellow spot virus (IYSV)	Nucleocapsid	Sanger sequencing	PZ715997	Iris yellow spot virus isolate IYSV-Lisianthus nucleocapsid-like (N) gene, partial sequence (HM035070.1)	100%

* Garlic latent virus (GLV) is recognized as synonymous with SLV (Shallot latent virus).

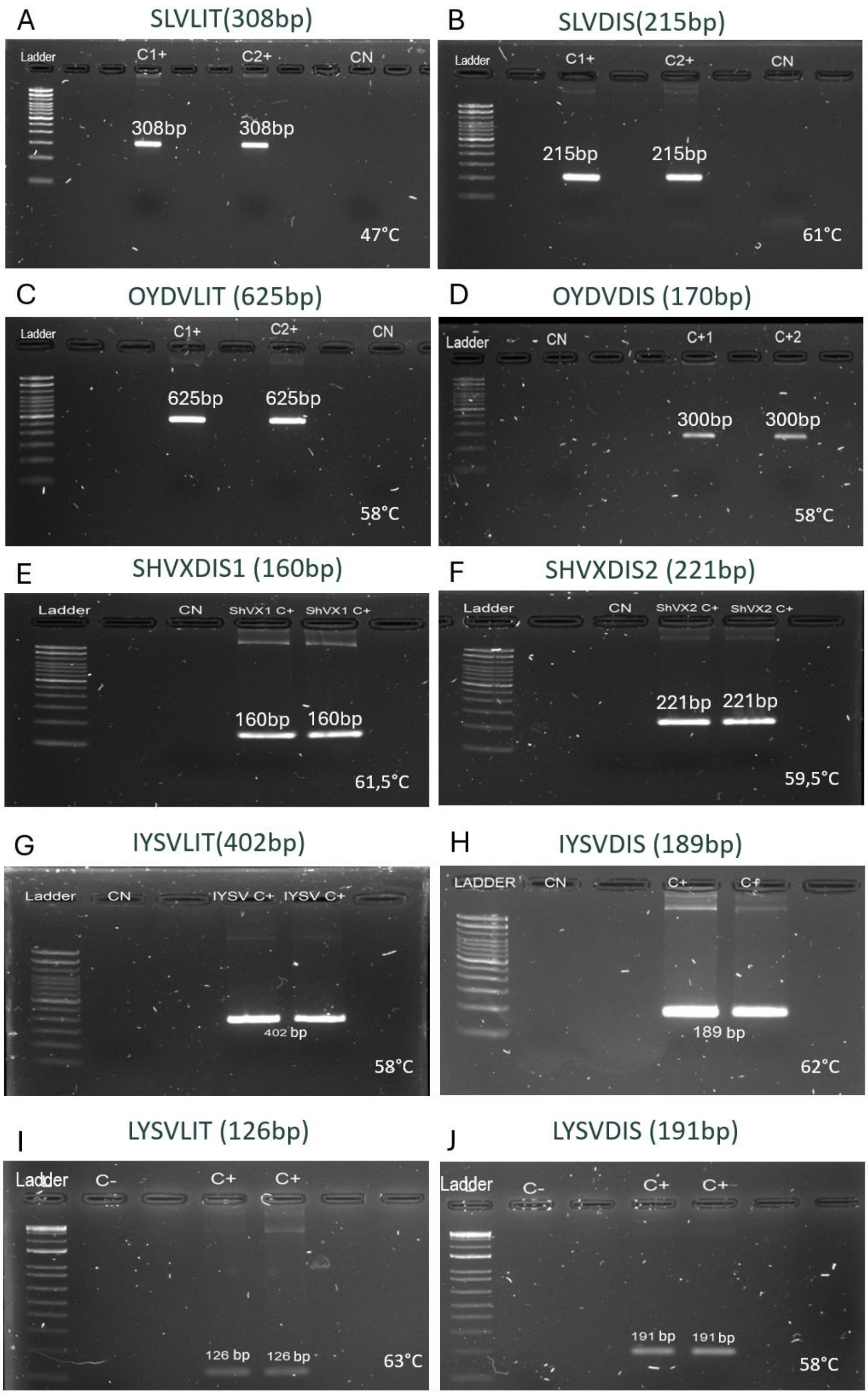

Figure S1. Amplification of PCR products of positive controls with designed and literature-based primers. (**A**) PCR products of SLVLIT with positive controls. (**B**) PCR products of SLVDIS with positive controls. (**C**) PCR products of OYDVLIT with positive controls. (**D**) PCR products of OYDVDIS with positive controls. (**E**). PCR products of ShVXDIS1 with positive controls. (**F**) PCR products of ShVXDIS2 with positive controls. (**G**) PCR products of IYSVLIT with positive controls. (**H)** PCR products of IYSVDIS with positive controls. (**I**) PCR products from LYSVLIT with positive controls. (**J**) PCR products of LYSVDIS with positive controls. The lack of a band implies the absence of the virus. Abbreviations: Ladder: ladder, C-: negative control, C+: positive control.

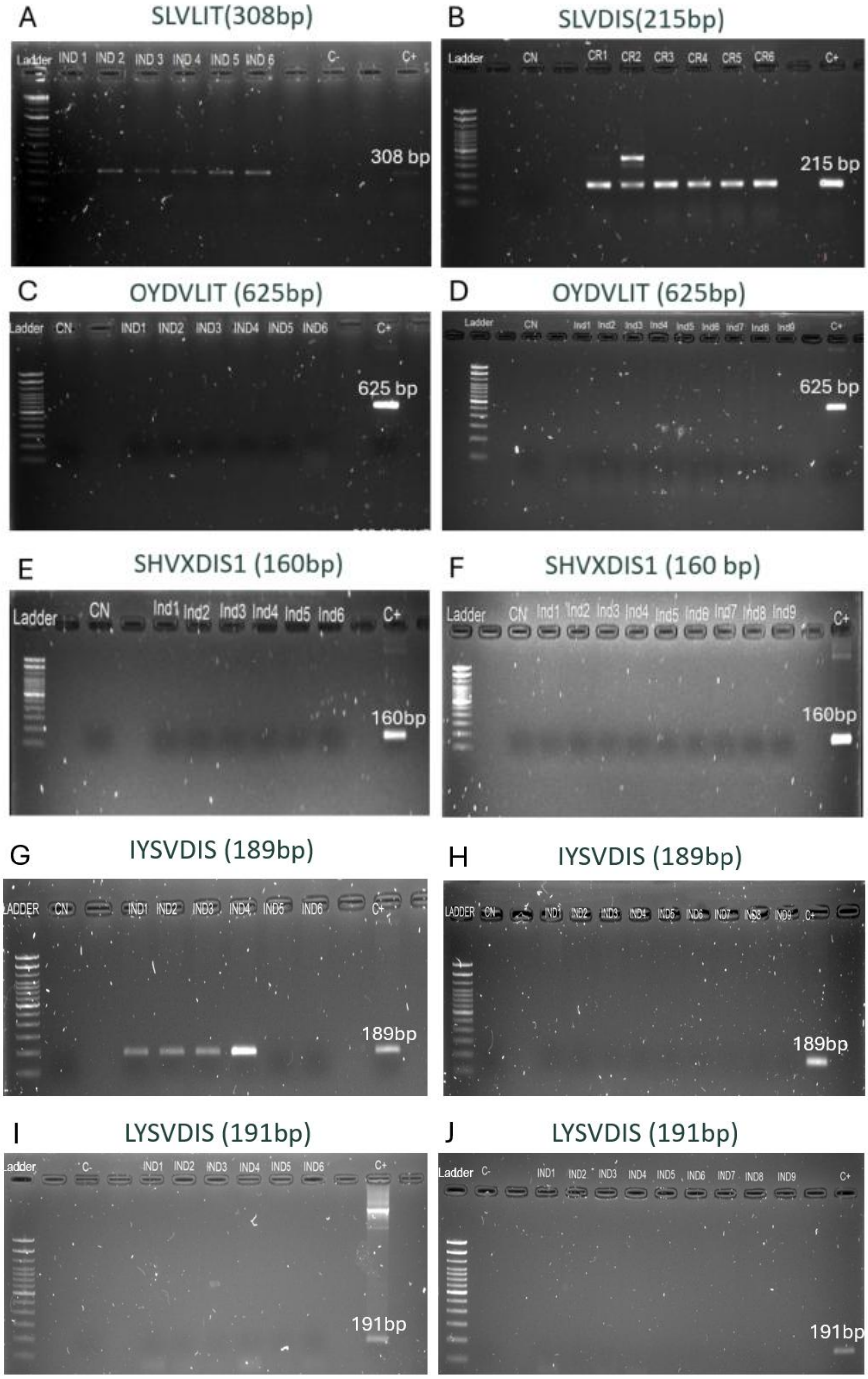

Figure S2. Pilot evaluation of PCR products of shallot samples with designed and literature-based primers. (**A**) PCR products of SLV with literature primers. (**B**) PCR products of SLV with designed primers. (**C, D**) PCR products of OYDV with literature primers. (**E, F**) PCR products of ShVX with designed primers. (**G, H**) PCR products of IYSV with designed primers. (**I, J**) PCR products of LYSV with designed primers. The lack of a band implies the absence of the virus. Abbreviations: L: ladder, C+: positive control.

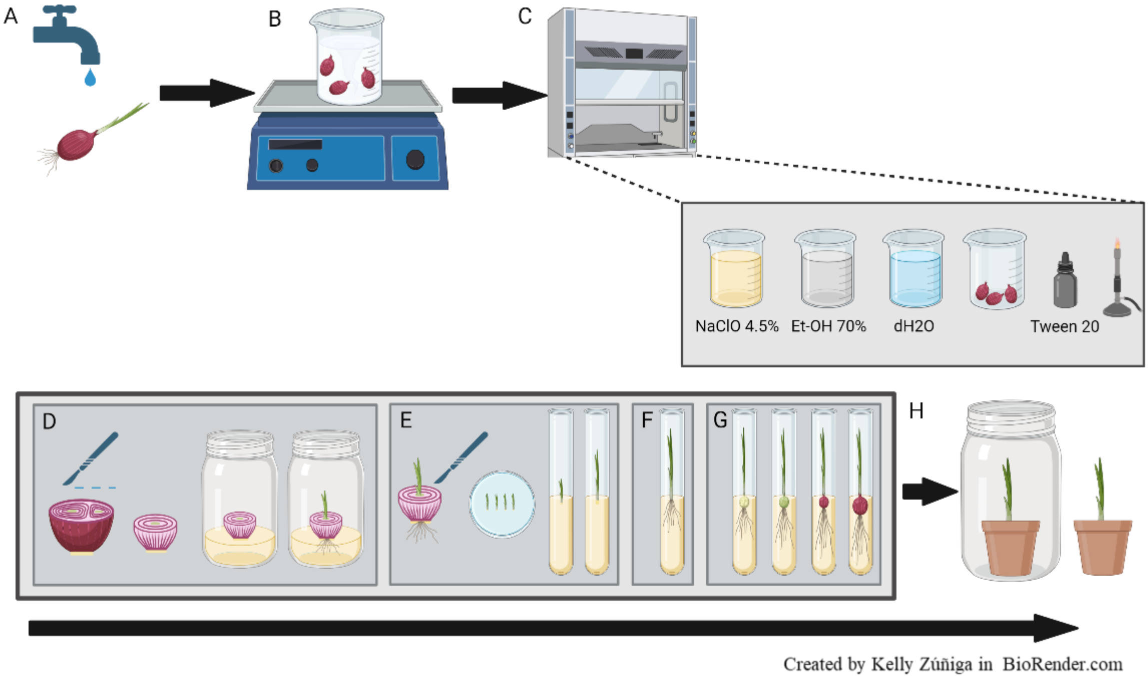

Figure S3. Scheme of shallot shoot regeneration from shoot tips. **A.** Washing bulbs with tap water. **B.** Washing bulbs with Mertect fungicide (15 minutes). **C.** Disinfection protocol with ET-OH 70%, NaClO 4.5%, and Tween 20 inside the laminar flow chamber. **D.** Culture of basal discs (M1 medium). **E.** Shoot tip culture (M1 medium). **F.** Rooting (M2 medium). **G.** Bulb formation (M3 medium). **H.** Acclimatization of shallots.

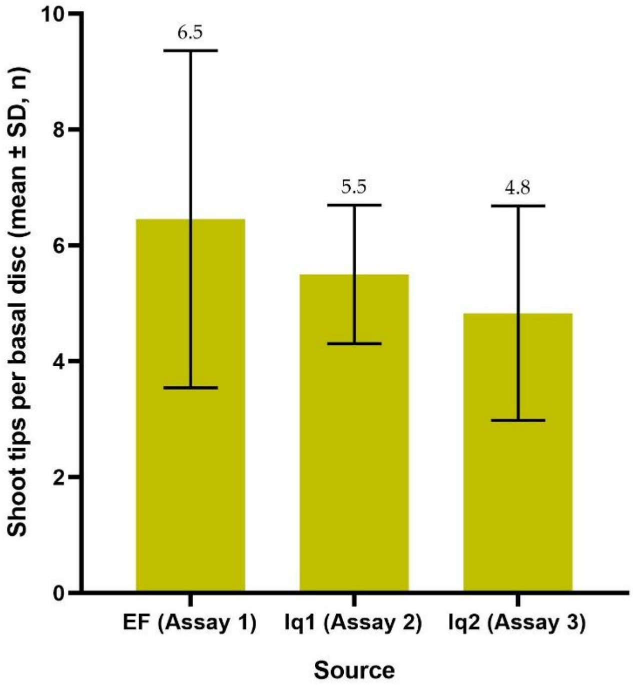

Figure S4. The mean number of shoots tips extracted per basal disc under different assays. Data represent mean ± SD (*n* = X). Statistical significance was assessed by one-way ANOVA ($p = 0.2466$). Abbreviation: EF, Experimental Farm; Iq1, Iñaquito 1; Iq2, Iñaquito 2.

References

1. Majumder S, Baranwal VK, Joshi S. Simultaneous detection of onion yellow dwarf virus and shallot latent virus in infected leaves and cloves of garlic by duplex RT-PCR. Journal of Plant Pathology. 2008;90: 371–374.

2. Mahmoud S, A. Abo-El S, M. El-Boro A, H. Abdel-G M. Identification of Onion yellow dwarf potyvirus as One of the Major Viruses Infecting Garlic in Egypt. International J of Virology. 2008;4: 1–13. doi:10.3923/ijv.2008.1.13

3. Leach A, Fuchs M, Harding R, Schmidt-Jeffris R, Nault BA. Importance of Transplanted Onions Contributing to Late-Season *Iris yellow spot virus* Epidemics in New York. Plant Disease. 2018;102: 1264–1272. doi:10.1094/PDIS-06-17-0793-RE

4. Katis NI, Maliogka VI, Dovas CI. Viruses of the Genus Allium in the Mediterranean Region. Advances in Virus Research. Amsterdam, The Netherlands: Elsevier; 2012. pp. 163–208. doi:10.1016/B978-0-12-394314-9.00005-1

5. Vega J, Arahana B. VS, Torres MDL. Estandarización de un protocolo de regeneración de cebolla chalote (Allium cepa var. aggregatum) a partir de meristemas apicales. 2015 [cited 10 Apr 2024]. doi:10.18272/aci.v7i1.225
